# Supplementary material for: Resolving the full spectrum of human genome variation using Linked-Reads
Source: Genome Res. 2019 Apr;29(4):635–45. doi: 10.1101/gr.234443.118 (PMC6442396; doi:10.1101/gr.234443.118)
Supplement: Supplemental Material [file supp_gr.234443.118_Supplemental_longranger-2.2.zip › longranger-2.2.tar.bz2!/lr2.2-public/lib/stan/cmdstan/stan/doc/api/html/functions_vars_m.html]

Stan: Class Members - Variables


|  |  |
| --- | --- |
|  | Stan  2.5.0  probability, sampling & optimization |


- Main Page
- Related Pages
- Namespaces
- Classes
- Files

- Class List
- Class Index
- Class Hierarchy
- Class Members

- All
- Functions
- Variables
- Typedefs
- Enumerator
- Related Functions

- \_
- a
- b
- c
- d
- e
- f
- g
- h
- i
- k
- l
- m
- n
- o
- p
- q
- r
- s
- t
- u
- v
- w
- x
- y
- z


All Classes Namespaces Files Functions Variables Typedefs Enumerations Enumerator Friends Macros Pages

### - m -

- M\_
  : stan::gm::cholesky\_factor\_var\_decl
  , stan::gm::matrix\_var\_decl
  , stan::gm::vector\_var\_decl
  , stan::math::coupled\_ode\_system< F, double, double >
  , stan::math::coupled\_ode\_system< F, double, stan::agrad::var >
  , stan::math::coupled\_ode\_system< F, stan::agrad::var, double >
  , stan::math::coupled\_ode\_system< F, stan::agrad::var, stan::agrad::var >
- map\_
  : stan::gm::variable\_map
- matrix\_decl\_r
  : stan::gm::var\_decls\_grammar< Iterator >
- max\_delta\_
  : stan::mcmc::base\_nuts< M, P, H, I, BaseRNG >
- max\_depth\_
  : stan::mcmc::base\_nuts< M, P, H, I, BaseRNG >
- max\_exponent
  : std::numeric\_limits< stan::agrad::fvar< T > >
  , std::numeric\_limits< stan::agrad::var >
- max\_exponent10
  : std::numeric\_limits< stan::agrad::fvar< T > >
  , std::numeric\_limits< stan::agrad::var >
- maxIts
  : stan::optimization::ConvergenceOptions< Scalar >
- metadata
  : stan::io::stan\_csv
- metric
  : stan::io::stan\_csv\_adaptation
- min\_exponent
  : std::numeric\_limits< stan::agrad::fvar< T > >
  , std::numeric\_limits< stan::agrad::var >
- min\_exponent10
  : std::numeric\_limits< stan::agrad::fvar< T > >
  , std::numeric\_limits< stan::agrad::var >
- minAlpha
  : stan::optimization::LSOptions< Scalar >
- mInv
  : stan::mcmc::dense\_e\_point
  , stan::mcmc::diag\_e\_point
- model
  : stan::io::stan\_csv\_metadata
  , stan::model::model\_functional< M >
- model\_
  : stan::mcmc::base\_hamiltonian< M, P, BaseRNG >
- model\_name\_
  : stan::gm::program\_grammar< Iterator >
- model\_r
  : stan::gm::program\_grammar< Iterator >
- msgs\_
  : stan::math::coupled\_ode\_system< F, double, double >
  , stan::math::coupled\_ode\_system< F, double, stan::agrad::var >
  , stan::math::coupled\_ode\_system< F, stan::agrad::var, double >
  , stan::math::coupled\_ode\_system< F, stan::agrad::var, stan::agrad::var >
- mu\_
  : stan::mcmc::stepsize\_adaptation


---

[ Stan Home Page ]

*© 2011–2014,
Stan Development Team.*
